# Supplementary material for: Use of Visual Pedagogy to Help Children with ASDs Facing the First Dental Examination: A Randomized Controlled Trial
Source: Children (Basel). 2022 May 16;9(5):729. doi: 10.3390/children9050729 (PMC9139454; doi:10.3390/children9050729)

LA MIA VISITA DAL DENTISTA

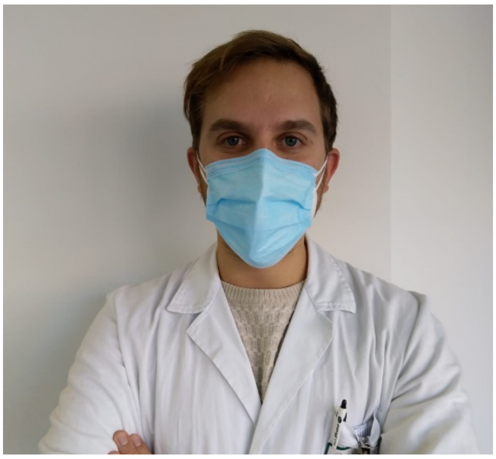

LUI È LUCA

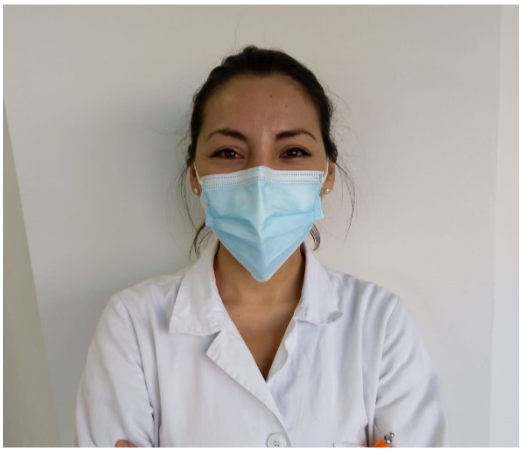

E LEI È LJUBICCA

SONO I DENTISTI CHE GUARDERANNO I TUOI DENTINI

COSA SUCCEDERA' QUANDO ANDRAI DAL DENTISTA?

- IN **SALA D'ASPETTO** ASPETTO SEDUTO IL MIO TURNO

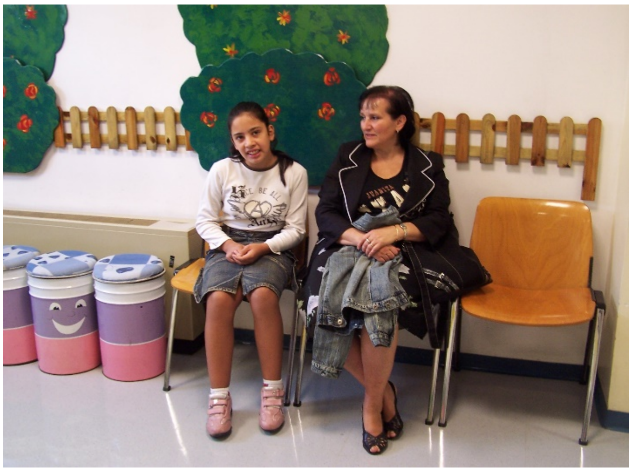

□ QUANDO IL DENTISTA MI CHIAMA **ENTRO**

□ MI SIEDO SULLA **POLTRONA** SPECIALE DEL DENTISTA, CHE SI ALZA E SI ABBASSA

□ IL DENTISTA MI METTE LA **BAVAGLIA**

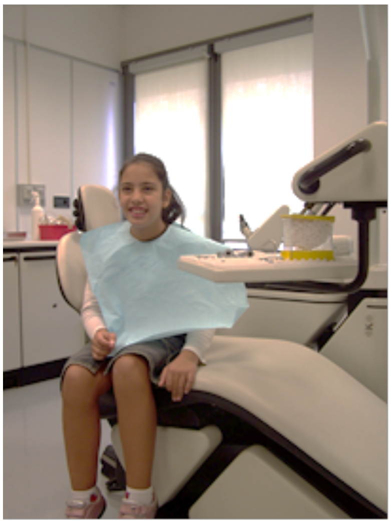

□ IL DENTISTA ACCENDE UNA **LAMPADA** PER VEDERE MEGLIO I MIEI DENTI

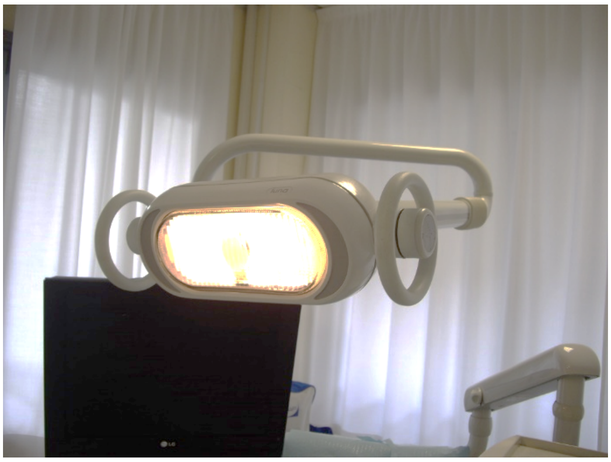

□ IL DENTISTA INDOSSA **GUANTI E MASCHERINA**

□ IL DENTISTA **CONTA I MIEI DENTI** E PER FARLO USA:

UNO SPECCHIETTO

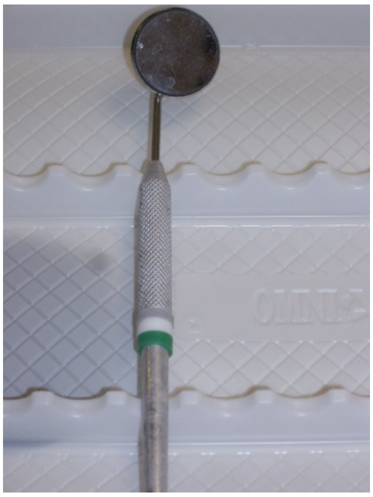

UNA MATITA

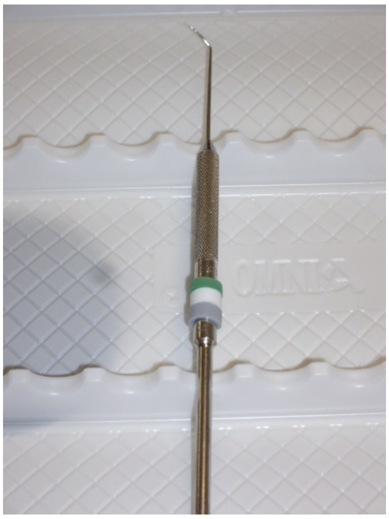

□ IO DEVO **APRIRE BENE LA BOCCA**

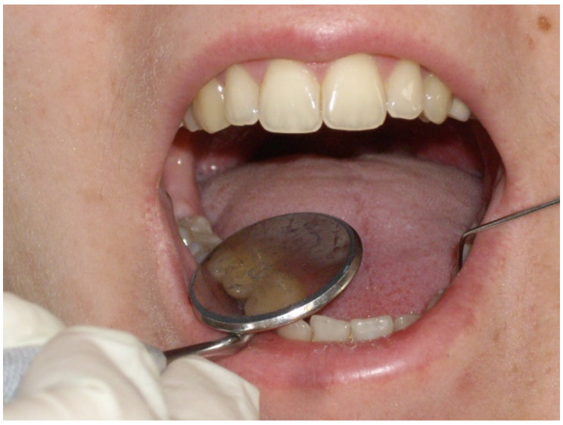

SONO STATO BRAVO! MI MERITO UN **PREMIO**

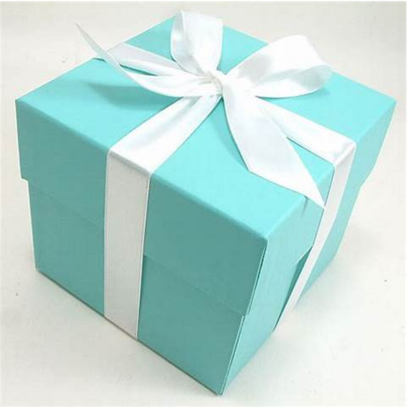

Supplement: Supplementary file 1 [file children-09-00729-s001.zip › File S2.pdf]
