# Supplementary material for: Use of Visual Pedagogy to Help Children with ASDs Facing the First Dental Examination: A Randomized Controlled Trial
Source: Children (Basel). 2022 May 16;9(5):729. doi: 10.3390/children9050729 (PMC9139454; doi:10.3390/children9050729)
Supplement: Supplementary file 1 [file children-09-00729-s001.zip › File S3.pdf]

ID	
Birth	

	Yes	No
**Do you think that the visit was a…… experience for your child?**		
Positive		
Negative		
**How much useful do you consider the visual aid provided to prepare your child at the visit?**		
Very useful		
Quite useful		
A bit useful		
Uneuseful		
