# Supplementary material for: Use of Visual Pedagogy to Help Children with ASDs Facing the First Dental Examination: A Randomized Controlled Trial
Source: Children (Basel). 2022 May 16;9(5):729. doi: 10.3390/children9050729 (PMC9139454; doi:10.3390/children9050729)
Supplement: Supplementary file 1 [file children-09-00729-s001.zip › File S4.pdf]

ID	
Birth	

	Yes	No
**Did you use or did the educator use visual aids to prepare your child for the visit?**		
**If NO, for which reasons?**		
My child is unable to understand it		
The images were not captivating for my child, he got bored quickly		
My child did not cooperate enough		
Lack of time		
The educator has not deemed them suitable and/or does not believe they can be helpful for the child		
**If YES, how long did the child use them?**		
One or less than once per week		
3-4 times per week		
1 time per day		
More than 1 time per day		
**If YES, with whom did the child use them?**		
Only with parents		
Only with the educator		
Both with parents and educator		
**Did you child have previously used visual aid during his educational path?**		
**Did you use other visual aids beside the one provided in order to prepare your child to the visit?**		
